# Effectiveness of Innovation Media for Improving Physical Distancing Compliance during the COVID-19 Pandemic: A Quasi-Experiment in Thailand

**DOI:** 10.3390/ijerph17228535

**Published:** 2020-11-17

**Authors:** Hattaya Chutiphimon, Apinya Thipsunate, Atigun Cherdchim, Bootsarakam Boonyaphak, Panat Vithayasirikul, Patiphan Choothong, Swit Vichathai, Pitchayanont Ngamchaliew, Polathep Vichitkunakorn

**Affiliations:** 1 Faculty of Medicine, Prince of Songkla University, Songkhla 90110, Thailand; 5910310167@psu.ac.th (H.C.); 5910310172@psu.ac.th (A.T.); 5910310168@psu.ac.th (A.C.); 5910310095@psu.ac.th (B.B.); 5910310104@psu.ac.th (P.V.); 5910310090@psu.ac.th (P.C.); 5910310154@psu.ac.th (S.V.); 2 Department of Family and Preventive Medicine, Faculty of Medicine, Prince of Songkla University, Songkhla 90110, Thailand; pitchayanont@hotmail.com

**Keywords:** COVID-19, physical distancing, innovation media

## Abstract

To flatten the curve of COVID-19 infections, with no effective pharmacological interventions or vaccine available in the imminent future, public health responses must continue to rely on non-pharmacological interventions. We developed three innovation media to promote physical distancing compliance (i.e., a fearful picture, a red one-way arrow sign, and a norm-speech sticker). This study aimed to compare physical distancing compliance between our interventions and conventional interventions. Our study was a quasi-experiment, and we observed a representative sample of university canteen customers via closed-circuit television (CCTV). Each intervention was monitored over non-prime-time hours, per day, on 6–9 August 2020. Among the 400 participants (100 participants in each group), their age group, gender, and physical distancing practices were observed in a university canteen. The number of failures of physical distancing ranged between 93.8% and 17.6%, and on average between 84.2% and 34.2%, dependent on the intervention and the marking point. There were no statistically significant differences in promoting physical distancing compliance between our interventions compared with conventional interventions. However, the participants tended to practice physical distancing at the back of the queue more than at the front, regardless of the interventions.

## 1. Introduction

COVID-19 has ultimately changed the world’s view of pandemics, with drastic consequences to global health and economies. Within six months (from January to June 2020), 210 countries and territories around the world have reported more than seven million confirmed cases including almost four hundred thousand deaths as of 8 June 2020 [1]. SARS-CoV2, a highly infective pathogen, is mainly transmitted via droplet spread, which requires close contact [2]. It causes moderate to severe clinical outcomes in about 20% of all recognized infected individuals. The pathogen’s effect on health, wellbeing, business, and other aspects of daily life are felt throughout societies as well as on an individual level. Its potential effects are leading to the risk of health-system collapse, due to the overwhelming medical resources required. In addition to the global healthcare-system crisis [3], it also contributes to the risk of economic losses. Further, COVID-19 itself, physical distancing measures, and the potential economic fallout evoke psychological distress [4]. With no effective pharmacological interventions or vaccine being available in the imminent future, reducing the transmission of infection is the first priority. Hence, public health responses will continue to rely on non-pharmacological interventions (e.g., hand hygiene, wearing a face mask, avoidance of face touching, and physical distancing) to flatten the curve of infection [5]. Consequently, by adopting these measures, health-care systems may be able to handle this ongoing pandemic with limited resources.

Many governments have adopted physical distancing measures to mitigate the impact of the COVID-19 pandemic. Since March 2020, the Thai government has implemented physical distancing measures including stay-at-home orders and the closure of schools, restaurants, and other public places, in addition to restricting the number of people allowed to socially gather. While non-pharmacological strategies minimize opportunities for the transmission of the pandemic disease [6,7], the prolongation of these measures causes societal and economic disruption [8]. Thus, individual physical distancing is the most effective way to return to daily life, in order to balance the competing risks of health-system collapse and economic fallout.

Given these considerations, previous studies on non-pharmacological interventions were conducted for their effectiveness and for factors affecting compliance. However, there have been few studies on physical distancing interventions and their effectiveness in Thailand during this ongoing COVID-19 pandemic. Although there have been similar methodological and conceptual studies, these studies are scarce in relation to physical distancing interventions.

Therefore, in this study, we created interventions based on psychological theory, including color psychology and fear arousal [9,10], which could encourage physical distancing compliance among the general population in the community. We evaluated whether these interventions might be sufficient to improve physical distancing compliance, by observing behavioral changes. The results would ultimately provide evidence to revise subsequent infectious disease prevention policies.

This study aimed to compare physical distancing compliance between our developed interventions (i.e., a fearful picture, a red one-way arrow sign, and a norm-speech sticker) and conventional interventions in a university canteen.

## 2. Materials and Methods

### 2.1. Study Design and Population

This study was a quasi-experiment with a comparative behavioral observation study. Our target population was focused on university canteen customers at the Prince of Songkla University. The sampling technique was obtained from the first 100 participants, starting at 11.00 a.m. and ending at 1.00 p.m. in order to minimize over- and underpopulation, which could have potentially confounded the results.

### 2.2. Data Collection and Instruments

The data collection method was physical distancing observation via closed-circuit television (CCTV) at the university canteen. We observed genders, age groups, companions, carried items, and the physical distancing practices of the participants. Three independent researchers collected the data and judged the physical distancing.

### 2.3. Dependent Variable: Physical Distancing

Physical distancing in this study was defined as at least a 1.0-m distance among people [11]. People defined as maintaining positive physical distancing followed these criteria:

1. Standing within the marking position during the process of queueing;
2. Moving out of the marking position for 3 s or less each time was acceptable.

Other conditions, which did not meet the criteria above, were defined as a failure to maintain physical distancing. These criteria were used in a pilot study, and the observers were trained to improve reliability. This study was designed to include customers who appeared at the setting during the period of data collection and excluded those who were employees working in the setting and dependent people (wheelchair-dependent people and customers using a crutch).

### 2.4. Independent Variable: Innovation Media

**Figure 1:**
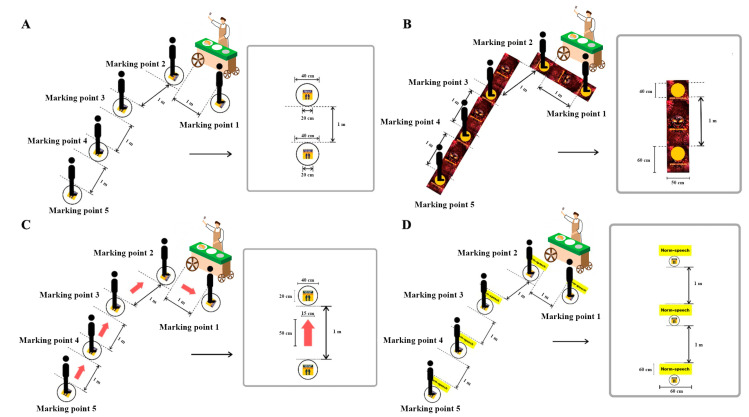
Innovation media: (**A**) Conventional, (**B**) fearful picture, (**C**) red one-way arrow sign, and (**D**) norm-speech sticker (e.g., “Please maintain a distance from other customers”, “Physical distancing and Win COVID-19”, and “Please queue here”).

We identified the university canteen as the experimental setting. Prior to our interventions, a footprint standing sign had been used, since July 2020, as the conventional intervention to indicate to customers how to practice physical distancing. We developed three other innovation media to promote physical distancing compliance:

1. The control or conventional sign was a footprint standing sign (Figure 1A);
2. A fearful picture, an aggressive COVID-19 with a punched-out circle, was the standing point (Figure 1B);
3. A red one-way arrow sign was placed between conventional interventions to instruct on direction (Figure 1C);
4. A norm-speech sticker was used to show speech that could encourage physical distancing compliance (Figure 1D), for example, “Please maintain a distance from other customers”, “Physical distancing and Win COVID-19”, and “Please queue here”.

No other physical distancing promotion activities were included as part of the intervention. The conventional intervention was monitored over a 2-h period per day (11.00 a.m. to 1.00 p.m.) on 6 August 2020. Then, our three developed interventions were implemented and observed on 7–9 August 2020, respectively.

### 2.5. Statistical Analysis

Data analysis included both continuous and categorized variables for physical distancing. The median and interquartile range (IQR) were used to describe physical distancing, as the data were not normally distributed. The Wilcoxon signed-rank test was used to compare physical distancing regarding different interventions within the same cluster. Categorical variables were analyzed using a Chi-square or Fisher’s exact test. Subgroup analysis by marking points was performed to minimize selection bias. The analysis was computed by R^®^ 4.0.0 (R core team, Vienna, Australia).

### 2.6. Ethics Statement

The study was conducted in line with the Belmont Report and was approved by the Human Research Ethics Committee (HREC), Faculty of Medicine, Prince of Songkla University (Ref. no: REC.63-272-9-1).

## 3. Results

### 3.1. Participant Characteristics

Among 400 participants (100 participants in each group), the characteristics of the participants were not different between a control, fearful picture, red one-way arrow sign, and norm-speech sticker intervention, except for the age group and carried items (Table 1). The most frequent age range was 19–64 years, 80.0% were not wearing university uniforms, 58.5% were female, 75.8% did not come with a companion, 50.0% carried item(s), and the most carried item was a bag (33.8%).

### 3.2. Physical Distancing and Innovation Media: Marking-Point Analysis

**Figure 2:**
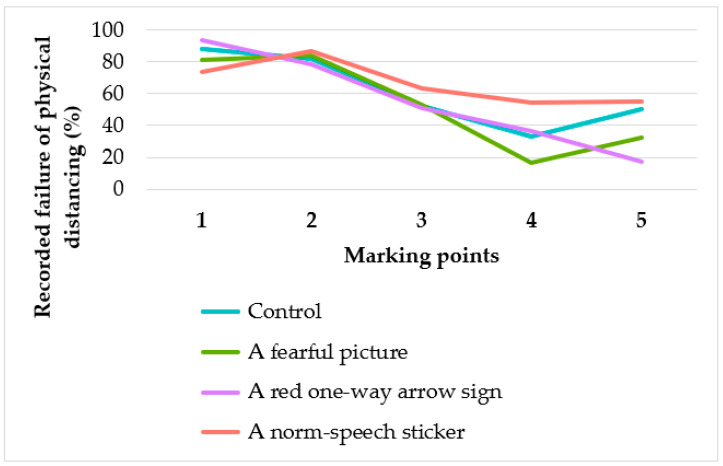
The percentage of recorded failures of physical distancing according to the marking point.

Analyses were performed according to 5 marking points; the results are provided in Table 2. The participants tended to fail to practice physical distancing at the 4th–5th (34.2–38.8%) less than at the 1st–3rd marking (84.2–55.2%) points (*p*-value < 0.001 for all interventions and total). At the 1st–2nd marking positions, approximately 80% of participants were considered to fail to practice physical distancing regardless of the intervention (Figure 2). For the norm-speech sticker, at the 1st marking position, the number recorded as failures of physical distancing was statistically significantly less than the conventional intervention (*p*-value = 0.02). For the fearful picture, the 1st, 4th, and 5th marking points had a lower proportion of failures of physical distancing compared with the control group, although these were not statistically significant. The red one-way arrow sign tended to result in the practice of physical distancing at the 2nd, 3rd, and 5th marking points. However, these differences were also not statistically significant.

## 4. Discussion

### 4.1. Summary of Results

Our developed interventions (i.e., fearful picture, red one-way arrow sign, and norm-speech sticker) in promoting physical distancing compliance were not statistically significantly different from the conventional intervention in the university canteen. In the subgroup analysis, there were fewer recorded failures of physical distancing than in the control group at some marking points.

Considering the level of self-awareness, which varies among people, this could potentially interfere with physical distancing compliance, due to the subgroup analysis data. Additionally, the consumptive behavior of people tends to result in buying decisions being made at the same places, which can cause repeat customers. However, we decided not to identify the participants due to confidentiality issues. As the 1st–3rd marking positions were along the counter, participants were distracted by menu selection, adding chili paste, and making online payments; thus, they tended to move out of the marking positions, leading to an overestimated number of recorded failures of physical distancing. Some participants also tended to buy items from the same restaurant repeatedly. The study was held during the period with zero new COVID-19 infections reported in Thailand so that the participants’ level of awareness was altered. Additionally, due to the uncertain number of customers, in cases of overpopulation, it was too crowded to maintain physical distancing, while underpopulation made it unnecessary to stand in the marking position. According to CCTV records, from 12.00 to 12.30 p.m. (prime-time hour due to lunch time), there was overpopulation. Hence, we decided to collect the appropriate number of customers during a non-prime-time hour. There was a higher proportion of children to adolescents and those who came with a companion in the control group, compared with other interventions (Table 1). This may overestimate the number of recorded failures to physical distance in the conventional group. However, our developed interventions still did not increase physical distancing practice compared with the control group.

### 4.2. Comparison with Prior Studies and Theoretical Frameworks

The results of promoting physical distancing compliance using the fearful picture were inconsistent with a study of protection motivation theory, which refers to the motivation to protect oneself against a health threat (i.e., the person tended to have a stronger intention to adopt the recommended action [10]). For the red one-way arrow sign intervention, the results were inconsistent with a study on encouraging handwashing among primary school students in Bangladesh, which increased handwashing to 58% of children, after foot paths connected latrines to handwashing infrastructure [12]. For the norm-speech sticker intervention, the conclusions were inconsistent with a study of experimental pretesting of handwashing interventions in a natural setting. It has been shown that normative messages can increase the handwashing ratio compared with control conditions [13].

In a study on the influence of self-awareness on human behavior by Robert A. Wicklund, people who have self-awareness are more likely to act consistently [14]. This agrees with a study stimulated by Duval and Wicklund’s self-awareness theory, in that self-focused attention influences a wide range of attitudes, attributions, and behaviors [15]. According to the current COVID-19 situation in Thailand, there have been no confirmed locally transmitted cases, which causes a reduction of self-awareness of precaution and prevention measures (e.g., hand hygiene, wearing a face mask, avoidance of face touching, and physical distancing) against COVID-19 infection among Thais. During the peak period of COVID-19 infections in Thailand, Thais strictly practiced physical distancing since they understood the severity of disease. On the other hand, in the period of no confirmed locally transmitted cases, physical distancing practices were reduced among Thai people, which is consistent with the stage of change theory [16,17].

### 4.3. Limitations and Strengths

Our study has a number of limitations. Firstly, our study was part of the family medicine course, which had a short timeline for performing this study. Thus, the interventions could not be employed on the same day of the week. For example, all interventions were completed on Mondays in order to minimize selection bias during the study, leading to the heterogenicity of the participants’ demographic data in each group. Secondly, data collection via CCTV records was time-consuming, which led to a small sample size for the subgroup analysis. An analysis of baseline characteristics (e.g., gender, age group, carrying items) could not be performed in this study. Thirdly, the participants were limited mainly to university students, who do not represent the general population, and the experimental setting was a canteen. Hence, it is too early to generalize our findings to a nationwide setting. Lastly, this study had an observational behavioral design. The explanation of how the participants failed to physical distance was limited.

Despite the limitations, our results were obtained via direct observation of human behavior in a natural setting. Therefore, they can represent actual human responses to our interventions. The results were considered to be a worthwhile beginning to improve prevention measures of disease transmission in the future. As there have been few studies conducted on physical distancing interventions and their effectiveness in Thailand during this ongoing COVID-19 pandemic, our study is one of the earliest studies to be conducted.

### 4.4. Implications and Further Studies

Some countries are still dealing with large COVID-19 outbreaks, but even those currently controlling the virus fear a second wave. Many countries (e.g., Vietnam and New Zealand) are seeing a resurgence in COVID-19 cases after successfully controlling its spread. Our interventions were simple and affordable to install and used locally available materials without additional training. Future studies involving a large number of participants would compare the behavioral impact of our interventions. Furthermore, including standing instructions (i.e., “Stand here”) could be a more understandable and/or another intervention to be studied. The norm-speech sticker should be placed at eye level. Additionally, exploring the long-term sustainability of behavioral impacts is also necessary. Henceforward, studies should be performed in appropriate settings with the fewest potential confounders. Lastly, other studies should retain more precise evaluation criteria of positive physical distancing practices.

## 5. Conclusions

This study has provided evidence that our interventions could not increase physical distancing practices compared with conventional interventions due to potential confounders and limitations. A combination of interventions could probably better motivate physical distancing practices among people. If possible, exploring the long-term sustainability of behavioral impacts is suggested.

## Figures and Tables

**Table 1:** Demographic data of the participants (*n* = 400).

Characteristics	Control (*n* = 100)	A Fearful Picture (*n* = 100)	*p*-Value	A Red One-Way Arrow Sign (*n* = 100)	*p*-Value	A Norm-Speech Sticker (*n* = 100)	*p*-Value
**Gender by observation, *n* (%)**							
Male	35 (35.0)	45 (45.0)	0.194 ^a^	37 (37.0)	0.883 ^a^	49 (49.0)	0.063 ^a^
Female	65 (65.0)	55 (55.0)		63 (63.0)		51 (51.0)	
**Wearing university uniform, *n* (%)**							
Yes (estimated 17–25 years)	2 (2.0)	35 (35.0)	<0.001 ^a,^*	17 (17.0)	<0.001 ^a,^*	26 (26.0)	<0.001 ^a,^*
No (estimated age group by observation)	98 (98.0)	65 (65.0)		83 (83.0)		74 (74.0)	
**Age group by observation, *n* (%)**							
Children (estimated 0–11 years)	1 (1.0)	0	0.002 ^b,^*	0	<0.001 ^b,^*	0	0.001 ^b,^*
Adolescences (estimated 12–18 years)	12 (12.0)	0		0		0	
Adults (estimated 19–64 years)	83 (83.0)	61 (61.0)		83 (83.0)		70 (70.0)	
Elderly (estimated ≥65 years)	2 (2.0)	4 (4.0)		0		4 (4.0)	
**With companion, *n* (%)**							
No	66 (66.0)	83 (83.0)	0.009 ^a,^*	75 (75.0)	0.215 ^b^	79 (79.0)	0.057 ^a^
Yes	34 (34.0)	17 (17.0)		25 (25.0)		21 (21.0)	
**Carrying item(s), *n* (%)**							
No	38 (38.0)	48 (48.0)	0.199 ^a^	65 (65.0)	<0.001 ^a,^*	49 (49.0)	0.154 ^a^
Yes	62 (62.0)	52 (52.0)		35 (35.0)		51 (51.0)	
Container	3	2		2		1	
Book	0	0		0		0	
Bag	44	37		26		28	
Mobile	26	31		10		26	
Other	7	3		3		10	
**Number of carrying item(s), *n* (%)**							
Median (IQR)	1 (1, 2)	1 (1, 2)	0.243 ^c^	1 (1, 1)	0.089 ^c^	1(1, 2)	0.596 ^c^
1	41 (66.1)	29 (55.8)	0.513 ^b^	29 (82.9)	0.156 ^b^	36 (70.6)	0.932 ^a^
2	19 (30.6)	20 (38.5)		5 (14.3)		14 (27.5)	
3	2 (3.2)	3 (5.8)		1 (2.9)		1 (2.0)	
4	0	0		0		0	

IQR = interquartile range, * *p*-value < 0.05, compared with control group, ^a^ Chi-square test, ^b^ Fisher’s exact test, ^c^ Wilcoxon Rank-sum test.

**Table 2:** Number of recorded failures of physical distancing at each marking point, compared with the control group.

Marking Point	Control	A Fearful Picture		A Red One-Way Arrow Sign		A Norm-Speech Sticker		Total
	No. of Failure/Total (%)	No. of Failure/Total (%)	*p*-Value ^a^	No. of Failure/Total (%)	*p*-Value ^a^	No. of Failure/Total (%)	*p*-Value ^a^	
1	87/99 (87.9)	77/95 (81.1)	0.264	91/97 (93.8)	0.234	70/95 (73.7)	0.020 *	325/386 (84.2)
2	81/99 (81.8)	83/99 (83.8)	0.851	78/99 (78.8)	0.721	86/99 (86.9)	0.434	328/396 (82.8)
3	22/42 (52.4)	38/72 (52.8)	1	32/63 (50.8)	1	45/71 (63.4)	0.341	137/248 (55.2)
4	8/24 (33.3)	9/55 (16.4)	0.164	12/33 (36.4)	1	25/46 (54.3)	0.156	54/158 (34.2)
5	6/12 (50.0)	13/40 (32.5)	0.317	3/17 (17.6)	0.106	16/29 (55.2)	1	38/98 (38.8)
*p*-value ^b^	<0.001 *	<0.001 *		<0.001 *		<0.001 *		<0.001 *

^a^ Chi-square test and compared with control group, ^b^ Chi-square test and compared within group, * *p*-value < 0.05.
